# A modelling study to explore the effects of regional socio-economics on the spreading of epidemics

**DOI:** 10.1007/s42001-024-00322-2

**Published:** 2024-08-14

**Authors:** Jan E. Snellman, Rafael A. Barrio, Kimmo K. Kaski, Maarit J. Korpi–Lagg

**Affiliations:** 1 Department of Computer Science, Aalto University School of Science, 00076 Aalto, Finland; 2 Instituto de Física, Universidad Nacional Autónoma de México, 01000 México D.F., Mexico; 3 The Alan Turing Institute, 96 Euston Rd, Kings Cross, London, NW1 2DB UK; 4 Max-Planck-Institut für Sonnensystemforschung, Justus-von-Liebig-Weg 3, 37077 Göttingen, Germany; 5 KTH Royal Institute of Technology and Stockholm University, Hannes Alfvéns väg 12, 11419 Nordita, Sweden

**Keywords:** Hybrid Epidemic Modelling, Agent-based Social Simulation, Machine Learning Assisted Data Analysis

## Abstract

Epidemics, apart from affecting the health of populations, can have large impacts on their social and economic behavior and subsequently feed back to and influence the spreading of the disease. This calls for systematic investigation which factors affect significantly and either beneficially or adversely the disease spreading and regional socio-economics. Based on our recently developed hybrid agent-based socio-economy and epidemic spreading model we perform extensive exploration of its six-dimensional parameter space of the socio-economic part of the model, namely, the attitudes towards the spread of the pandemic, health and the economic situation for both, the population and government agents who impose regulations. We search for significant patterns from the resulting simulated data using basic classification tools, such as self-organizing maps and principal component analysis, and we monitor different quantities of the model output, such as infection rates, the propagation speed of the epidemic, economic activity, government regulations, and the compliance of population on government restrictions. Out of these, the ones describing the epidemic spreading were resulting in the most distinctive clustering of the data, and they were selected as the basis of the remaining analysis. We relate the found clusters to three distinct types of disease spreading: wave-like, chaotic, and transitional spreading patterns. The most important value parameter contributing to phase changes and the speed of the epidemic was found to be the compliance of the population agents towards the government regulations. We conclude that in compliant populations, the infection rates are significantly lower and the infection spreading is slower, while the population agents’ health and economical attitudes show a weaker effect.

## Introduction

When facing an epidemic, human societies react in different ways trying mitigate its effects However, the measures the local governmental bodies take measures aiming to limit the spreading of an infectious disease can have undesirable societal consequences, such as social isolation and diminished economic activities. As these socio-economic concerns shape the epidemic response, the result is a feedback loop in which the societal reactions affect the epidemic spreading and vice versa.

The responses of human societies to large-scale crises are strongly guided by their prevailing societal values. For example, the responses may be influenced by how much social responsibility toward others and economic activity are valued. Recently, we have presented a proof of concept model combining socio-economic simulations with epidemic spreading, called the BTH-SEIRS model [1]. The epidemic part of this hybrid model is based on a SEIRS system[2–4] and it takes into account separately the properties of the disease, namely its virulence and lethality with stochastic mechanisms for the geographical spread by the population mobility[5–8], while the socio-economic part is based on an agent–based implementation of the better–than hypothesis (BTH), introduced in [9, 10] that governs how different agents and their values influence the spreading speed of the epidemic. The main motivation of the model was to create a rudimentary bridge between epidemic modeling and simulating the behaviour of human societies with a simple psychological model.

The BTH–SEIRS model contains two different types of agents, the population and government agents, characterised by a total of six parameters, each having an effect on the epidemic spreading [1], such that the populations value their own efforts to mitigate the epidemic, their health status and their compliance with the restrictions put in place by governments, while the governments value the health and economic situations in their districts. Of these parameters the compliance of the population turned out to have most impact on the spreading of the epidemic, with low or negative compliance leading to the epidemic spreading very fast in wavelike patterns, while high compliance resulted in the epidemic spreading slowly and chaotically. However, as these findings were based on relatively few simulations, they raised more questions, such as to what regions of the parameter space these different types of behaviour exist, how wide are the transitional regions between them, what is the possible transitional behaviour like, and whether completely new kinds of behaviour can be found in the six dimensional parameter space. In this study we set out to answer these questions.

In here, we write the equations in a different form to enable us to reduce the dimensionality of the parameter space from six to four. and with this scheme we performed a total of $10^5$ simulations in order to to adequately map the parameter space. These simulations produced a vast amount of data, and the analysis had to be automated.The analysis of the most prominent patterns in the data was carried out by principal component analysis (PCA [11–13]), self-organising maps (SOM [14]) and silhouette numbers [15]. Our aim is to determine whether the feature vectors formed from the simulation data exhibit clusters, whether they are significant, and which cluster has the most desirable property of slow infection spreading.

To our knowledge, the BTH–SEIRS-model is currently the only agent–based epidemic model, where the societal values of agents play a role in how they deal with the epidemic in a country wide scale. More specifically, we do not know any other agent–based model, in which the simulated agents themselves determine their own levels of effort to mitigate the effects of the epidemic according to their values. There are, of course, other approaches in agent–based epidemic modelling, summaries of which can be found in [16, 17]. The models most analogous to ours are those that study the effects of epidemics spreading in social networks in which agent behaviour is a factor [18–20]. Some of the agent-based models concentrate on the economic effects of epidemics, but these are usually oriented toward testing the effects of different mitigation measures such as non-pharmaceutical interventions (NPIs) [21–23], or the propagation of the effects of the epidemic via supply chains [24–26] rather than allowing the agents themselves determine the mitigating measures.

The rest of this paper is organised as follows: In the Methods–section we present the model, the parameter space and how the simulations are analysed. In the Results–section we concentrate on the main findings, while we relegate some of the more mundane results to an appendix. The significance of the results are discussed in the final section.

## Motivation

This study is an extension of earlier work [1], where we presented a novel hybrid epidemic model with agent–based and compartmental features. To summarize, in the model the simulated epidemic spreads through a two dimensional grid of geographic cells, each of which has a population whose epidemic situation is modeled by a compartmental SEIRS model. Epidemic spreads from one cell to others with a probability that is affected by the agent–based features of the model: The populations of the cells and the authorities of areas consisting of a number of cells can choose to make an economic contribution to mitigate the epidemic, i.e. lower the probability of its spread. These agents make their choices according to their values and naturally agents prioritising economic activity tend to choose lower mitigation measures than agents prioritising public health, with corresponding consequences for the evolution of the epidemic. The inspiration for our model was the public discourse surrounding COVID-19 pandemic in its early stages, during which it was loudly debated whether the pandemic mitigation measures were worth their economic and social costs. The purpose of the BTH–SEIRS model is to serve as a very simple depiction of the ways social values can influence the actions affected societies take toward epidemic mitigation and the influence of these actions to the evolution of the epidemic.

BTH–SEIRS model has a total of six different parameters representing the values the agents can have, requiring huge amounts of simulations to map out the wholeparameter space. This was not attempted yet in [1].We merely demonstrated its basic behaviours in a few instructive cases. In particular, we focused on different spreading patterns that we could readily find and their effects on the evolution of the epidemic and socio–economic effects measured by infection rated and agent mitigation measures, respectively. The easily recognisable spreading patterns were slow and chaotic, fast and wavelike, and an apparently transitional state between these with features of both. The purpose of the present study is to perform the desirable full parameter scan, classify the basic and possibly new behavioural patterns emerging with machine learning tools, namely self-organising maps (SOMs) and principal component analysis (PCA). Particularly, we aim at finding clusters of value parameter combinations that result in slow versus fast spread of the epidemic. This is our contribution to the emerging field of the machine learning assisted data analysis, a review of which can be found in [27].

## Methods

In [1] we presented an epidemic model with agent-based and geographical elements that we named the BTH–SEIRS model. The main ideas behind the model are that the actions of the populations affected by an epidemic have a feedback effect on the evolution of the epidemic itself, and that the actions of the populations are determined by the prevailing societal values. In this study we aim to investigate the effects of the values further in the BTH–SEIRS model. To this end, we varied the parameters representing the societal values in the model. Since the value parameter space of the model is quite large, we had to perform a huge number of simulations and analyse their results in mass scale using computational methods of the self organising maps (SOMs) and the principal component analysis (PCA). In the first part of this section we present the BTH–SEIRS model following very loosely the format of the ODD protocol [28] and in the second part the computational tools.

### Mathematical model

#### Purpose

The purpose of the BTH–SEIRS model is to describe a situation where an epidemic spreading through a populated geographical region inspires the affected populations to mitigate its spread. The motivation for the model is to simulate the feedback loop between the actions of the populations reacting to the epidemic and the evolution of the epidemic itself, of particular interest is the effect that societal values have on the behaviour of the population.

#### Entities, state variables and scales

The BTH–SEIRS model is a hybrid epidemic model which consists of an with agent based model (BTH) and a multiple compartmental SEIRS model on a geographical two dimensional space. The simulated geographical region is a two dimensional grid of cells. In each cell one defines SEIRS dynamical model. The cells are grouped together to form larger districts that represent administrative divisions of the region. The populations of the grid cells and the authorities of the districts are treated as agents, which can mitigate the spread of the simulated epidemic. The internal state variables of these agents can be divided into those related to their decision making algorithm driven by the BTH part and those related to the SEIRS part governing the evolution of the epidemic. For the population agents the former consists of the effort they put in mitigating the epidemic (*x*) and the latter of the proportions of the full population in the susceptible (*S*), exposed (*E*), infected (*I*) and recovered (*R*) compartments. The only state variable that the authority agents have is the restrictions (*X*) they impose on the population agents. Derived variables depicting the compliance of the population agents, the infection rates and the economic activity of the districts and the probability for the epidemic to spread from one cell to a neighbouring one are also important to the running of the model as detailed below. In this study, there is no meaningful spatial scale, since the spreading of the epidemic is stochastic, but the scale of the time-steps is one day.

#### Basic assumptions

Basic assumptions of the BTH–SEIRS model can be grouped into the assumptions regarding spreading and evolution of the simulated epidemic and the decision making of the population and authority agents. When it comes to the spreading of the epidemic from an infected geographical cell to an uninfected one we assume that the spreading process is stochastic, and occurs with a probability that is determined by the mobility of the simulated populations of the cells. Once the epidemic has spread to a cell, the evolution of the epidemic in that cell is assumed to be independent of the epidemic situation in other cells. In other words, we assume that the mobility of people only affects how the epidemic spreads from one area to another but otherwise has negligible effects on the infection numbers once the epidemic is in full swing. The evolution of the epidemic in each infected geographical cell is governed by a SEIRS compartmental model, in which people move from the susceptible compartment to exposed exposed, infective and recovered compartments, in that order, before eventually returning to the susceptible compartment.

The basic assumption of the BTH part of the model is that the simulated agents compare themselves to others of their type, and their main motivation is to attain as high relative social position as possible. A corollary of this assumption is that the values of the agents can be represented as comparison scales with associated weight parameters that determine how important it is for a given agent to rank high (if the weight parameter is positive) or low (if the weight parameter is negative) on each individual comparison scale. The weight parameters are in the context of BTH called value parameters. The comparison scales that govern the behaviour of the population agents are assumed to be the strength of their epidemic mitigation efforts, the infection rates and their compliance with the recommendations of the authority agents, while the comparison scales that govern the behaviour of the authority agents are their restrictions, total infection rates in their districts and the reduction of the economic activity in their districts. We also assume that the agents will take minimum mitigation efforts allowed by the BTH–utility function, which is detailed below.

In addition to the assumptions associated with the BTH and SEIRS parts of the model, we also assume that the mitigation measures of the population agents have a linear relation to the probability of the epidemic spreading from one geographical cell to others.

#### Evolution of the epidemic in a single cell: SEIRS

The SEIRS model has the following set of parameters that govern the epidemic development in each cell of the two dimensional grid at each timestep:

1. the period of latency before those who contract the disease become infectious $\epsilon$
2. the period of infectiousness $\sigma$
3. the period of immunity $\omega$
4. the mortality rate $\mu$
5. the transitivity of the disease $\beta$.

The dynamics of s of susceptible (S), exposed (E), infected (I) and recovered (R), are governed by the following map:

1 $$\begin{aligned} S_{t+1}&= q (S_t - G_t + \lambda q^b G_{t-1-b} ) + \mu N \nonumber \\ E_{t+1}&= q (E_t + G_t - q^{\epsilon } G_{t-\epsilon } ) \nonumber \\ I_{t+1}&= q (I_t + q^{\epsilon } G_{t-1-\epsilon }-q^b G_{t-1-a} ) \nonumber \\ R_{t+1}&= q (R_t + q^a G_{t-1-a}-q^b G_{t-1-b} ), \end{aligned}$$

where,

2 $$\begin{aligned} q &= 1 - \mu \nonumber \\ a& = \epsilon + \omega \nonumber \\ b&= \epsilon + \sigma + \omega \nonumber \\ G_{\alpha }&= S_{\alpha } (1 - e^{-\beta I_{\alpha }}), \end{aligned}$$

and $\lambda$ is the portion of the population that is susceptible again after being recovered. When a cell is infected at time $t_0$ the number of infected people is reset by the parameter $\eta$, such that $S_{t_0} = 1 - \eta$ and $I_{t_0} = \eta$.

#### Spreading of the epidemic between cells

The dynamics of epidemic is described by the SEIRS model introduced in [5], in which the epidemic could spread from one cell to a neighboring cell though a Monte Carlo process with probability given by a mobility parameter $v^t$, or to a more distant cell with some other probability to mimic different means of transportation e.g. air, rail, or roads. However, for the sake of clarity, long distance spreading is not considered in this study.

#### Coupling between the two parts of the model

The coupling between the grid cell populations and district authorities of the BTH model is accomplished by the dependence of the mobility $v_i^t$ on the BTH *x* variable. For the sake of simplicity, this dependence is assumed to be linear:

3 $$\begin{aligned} v_i^t = v_0 - x_i v_{max}, \end{aligned}$$

where $v_0$ is the maximum value of the mobility parameter, $0 \le x_i \le 1$ is the reduction of the socio-economic activity of the population agent *i*, and $v_{max} < v_0$ is the maximum reduction in the infectiousness of the disease that the socio-economic measures can deliver, since we assume that the population agents will not reduce their economic activity below the level necessary for their survival. For the purposes of this study we have arbitrarily set the base spreading probability $v_0$ to 0.5 and maximum reduction achievable with non–pharmaceutical interventions (NPIs) $v_{max}$ to 0.49.

#### Decision making of the agents: BTH

The behaviour of the agents are based on the following utility equations

4 $$\begin{aligned} u_i &= w^x_i \left(x_i + \sum _{j \in e_i} (x_i - x_j)\right) + w^y_i \left(y_i + \sum _{j \in e_i} (y_i - y_j)\right) + w^c_i \left(c_i + \sum _{j \in e_i} (c_i - c_j)\right), \end{aligned}$$

5 $$\begin{aligned} U_i &= W^X_i \left(X_i + \sum _{j \in E_i} (X_i - X_j)\right) + W^Y_i \left(Y_i + \sum _{j \in E_i} (Y_i - Y_j)\right) + W^Z_i \left(Z'_i + \sum _{j \in E_i} (Z_i - Z_j)\right), \end{aligned}$$

where the lower case letters refer to the population agent measures and capital letters to authority agent measures. Thus,

- $u_j$ and $U_k$ are the BTH utilities of the population agent *j* and the authority agent *k*, respectively.
- $x_j$ and $X_k$ are the quantities describing the reduction of economic activity by the population agent *j* and of the restrictions put in place by the authority agent *k*, respectively.
- $y_j$ and $Y_k$ are the quantities for infection rates in the cell of population agent *j* and the overall infection rated in the district governed by authority agent *k*, respectively.
- $c_j = x_j - X^r_j$ and $Z_k = \sum _{j \in D_k } x_j$ are the compliance of the population agent *j* and the overall reduction of economic activity in the district $D_k$ governed by authority agent *k*, respectively. $Z_k$ is linked to the gross domestic product $Z'_k$ by $Z'_k = 1 - Z_k$.
- $w^x_i$, $w^y_i$ and $w^c_i$ are the parameter values that the population agent *i* holds for their own effort to mitigate the epidemic, their own health and their compliance with the authorities’ restrictions, respectively.
- $W^X_i$, $W^Y_i$ and $W^c_i$ are the parameter values that the authority agent *i* holds for the restrictions they put in place to mitigate the epidemic and the overall health and economic activity in their districts, respectively.

With some assumptions, principally that the agents will make the minimum effort to mitigate the epidemic ($u_i=U_i=0$), we derived the following expressions for $x_i$ and $X_i$ for each time step:

6 $$\begin{aligned} x_i= & \frac{1}{|e_i| + 1} \left( \frac{w^x_i}{w^x_i + w^c_i} \sum _{j \in e_i} x_j - \frac{w^y_i}{w^x_i + w^c_i} (y_i + \sum _{j \in e_i} (y_i - y_j)\right) + \frac{w^c_i}{w^x_i + w^c_i} (|e_i| X^r_i - \sum _{j \in e_i} c_j), \nonumber \\ X_i= & \frac{1}{|E_i| + 1} \left(\sum _{j \in E_i} X_j - \frac{W^Y_i}{W^X_i} (Y_i + \sum _{j \in E_i} (Y_i - Y_j)\right) - \frac{W^Z_i}{W^X_i} (Z_i + \sum _{j \in E_i} (Z_i - Z_j))). \end{aligned}$$

#### Process overview

The processes of the model over one timestep take place in the following order:

1. Updating the sizes of the SEIRS compartments in each of the geographical cells where the epidemic has spread.
2. Simulating the spread of the epidemic. The epidemic spreads from one infected cell to another with a probability that is determined by the mitigating efforts of the population agents, as described above.
3. Update the mitigating efforts of the population agents and the restrictions imposed by the authority agents.

#### Emergence

If the model parameters are set to allow for a wide range of possible values for the spreading probability and the effect of the value parameters is observed, the simulated epidemic can be seen to spread with varying speed in accordance with the population agent mitigation measures. In our earlier studies we have determined that the value the population agents place on compliance with the restrictions of the authorities have the most noticeable effect (compliant populations are more effective in mitigating the spread), but other values have their effects as well.

#### Additional notes on the agent functions

The simulated agents have no adaptive, predictive or learning features. The population agents sense the infection rates and the mitigating efforts of the neighbouring population agents, while the authority agents sense the infection rates in their own areas and the restrictions enacted by all the other authority agents. The simulated agents do not interact directly, but the decisions they make on the mitigation efforts or restrictions have an effect how others behave.

### Data analysis methods and the surveyed parameter space

#### Surveyed parameter space and simulated data

As described above, the BTH–SEIRS model has a total of six different value parameters, which makes the value parameter survey a large computational task. However, it is possible to simplify the governing equations of the agents in a way that reduces the amount of value parameters by two. Since the minimum effort assumption implies $w^c_i < 0$ and $W^X_i < 0$, the equations 6 can be written in the form

7 $$\begin{aligned} x_i= & \frac{1}{|e_i| + 1} ( \frac{1}{\tilde{w^c_i} - 1} \sum _{j \in e_i} x_j - \frac{\tilde{w^y_i}}{\tilde{w^c_i} - 1} (y_i + \sum _{j \in e_i} (y_i - y_j))) + \frac{\tilde{w^c_i}}{\tilde{w^c_i} - 1} (|e_i| X^r_i - \sum _{j \in e_i} c_j), \nonumber \\ X_i= & \frac{1}{|E_i| + 1} (\sum _{j \in E_i} X_j + \tilde{W^Y_i} (Y_i + \sum _{j \in E_i} (Y_i - Y_j)) + \tilde{W^Z_i} (Z_i + \sum _{j \in E_i} (Z_i - Z_j))), \end{aligned}$$

where

8 $$\begin{aligned} \tilde{w^y_i} = \frac{w^y_i}{|w^x_i |}, \tilde{w^c_i} = \frac{w^c_i}{|w^x_i |}, \tilde{W^Y_i} = \frac{W^Y_i}{|W^X_i |}, \tilde{W^Z_i} = \frac{W^Z_i}{|W^X_i |}, \end{aligned}$$

and $w^c_i \ne w^x_i$ (or $\tilde{w^c_i} \ne 1$) always to prevent division by zero.

The formulations in Eqs. 7 and 8 allow us reduce the dimensionality of this parameter survey from six to four, since we need only consider the tilde parameters. Thus we conduct the survey with the following grid:

- $$\tilde{w^y_i} = -0.25, -0.5, -1, -2, -4,-3, -5, -10, -25, -50, -15, -20, -30, -40, -45$$
- $$\tilde{W^Y_i} = -0.25, -0.5, -1, -2, -4,-3, -5, -10, -25, -50, -15, -20, -30, -40, -45$$
- $$\tilde{w^c_i} = -0.25, -0.5, -1, -2, -4, 0.25, 0.5, 1, 2, 4, -0.75, -3, -5, -7.5, -10, 0.75, 3, 5, 7.5, 10$$
- $$\tilde{W^Z_i} = -0.25, -0.5, -1, -2, -4, 0.25, 0.5, 1, 2, 4, -0.75, -3, -5, -7.5, -10, 0.75, 3, 5, 7.5, 10$$

Here we have chosen $\tilde{W^Y_i}$ and $\tilde{w^y_i}$ to be always negative because of the assumption that the population and authority agents will make minimum efforts to mitigate the epidemic, ruling out herd immunity approach to dealing with the epidemic. We have chosen $\tilde{W^Z_i}$ to have both positive and negative values, even though it means that authority agents in the former case consider a declining economy to be an asset. This choice was made because there are no theoretical reasons to exclude positive values of $\tilde{W^Z_i}$, although there may be no real world authorities taking this view.

For each of these grid points we made two simulations in which the value parameters were randomized within the radius of 0.1 and the mean value indicated by the grid point, for a total of $10^5$ simulations. From these simulations we saved the information of the number of districts, size of the districts, value parameters of the authority and population agents, the times series of gross domestic products, government regulations, population compliance, infection rates by district, and finally the epidemic arrival time map $A_{ij}$ and the slowness $\Lambda$.

Of these the $A_{ij}$ and $\Lambda$ are measures of the speed of the spreading epidemic in the simulation, which we have introduced to monitor the spread of the epidemic with more detail in our simulations. These work as follows: The time step at which the epidemic spreads to geographical cell located at (*i*, *j*) is recorded to the arrival time map element $A_{ij}$. If the epidemic does not spread to a cell during the time frame of the simulation, the time will be marked down as the last time step of the simulation. The elements of the matrix $A_{ij}$ are then summed together to get an overall measure for the spreading rate of the epidemic,

9 $$\begin{aligned} A_{T} = \sum _{i,j} A_{ij}. \end{aligned}$$

$A_T$ attains its maximum value $A^{max}_T$ in the situation where the epidemic is not spreading at all from its initial beginning cell, in which case

10 $$\begin{aligned} A^{max}_T = T_s \times (N_c - 1) + 1, \end{aligned}$$

where $T_s$ is the simulation length and $N_c$ the number of simulated cells. Comparing $A_T$ to $A^{max}_T$ yields a fractional measure that describes the slowness of the epidemic spread

11 $$\begin{aligned} \Lambda = \frac{A_T}{A^{max}_T}, \end{aligned}$$

which we will call the $\Lambda$ measure. The lower $\Lambda$ is, the faster the propagation of the epidemic, with a theoretical minimum value of

12 $$\begin{aligned} min(\Lambda ) = \frac{N_c}{A^{max}_T}, \end{aligned}$$

which occurs in the case where the epidemic spreads instantly to all the geographical cells in a simulation, while $\Lambda = 1$ indicates that the epidemic does not spread at all, as stated above. The $\Lambda$ measure can be defined for each district separately by taking into account the arrival time of the epidemic into each district.

#### Data analysis methods

The quantities tracked by our model are the average economic activity, government regulations, dynamic compliance, infection rates in the nine modeled districts, and the $\Lambda$ measures defined in the previous section, and we construct feature vectors for classification purposes for all of these. With the information from the simulations we then endeavour to find different behavioural types in the simulations with self organising maps (SOM) using the Minisom python package [29]. We also made use of the Scikit Learn python package [30] to perform a principal component analysis (PCA)for the Minisom feature vectors for visualisation purposes.

Our data analysis workflow can be detailed as follows: For the first four measures listed above we construct the feature vectors by averaging the simulation data over time and then arranging the these averages by the district number, such that the feature vectors take the form (average measure in district 1, average measure in district 2,..., average measure in district 9). For the $\Lambda$ measure we take into account both the last $\Lambda$ time stamp in each district and the individual district $\Lambda$ measures and arrange this data into the following feature vector: (maximum recorded $\Lambda$ time step in district 1,..., maximum recorded $\Lambda$ time step in district 9, $\Lambda$ measure in district 1,..., $\Lambda$ measure in district 9). Next, we perform a dimensionality reduction on these vectors using PCA to visualise them in three dimensions to make sense of the classifications of this data for which purpose we use the SOMs. They require a two dimensional neuron matrix as input, to which the data is mapped. Here we use $1 \times n$ row matrices as the input, with the neuron counts *n* going from 2 to 20.

Having created these 19 different classifications for each of the feature vectors we proceed to evaluate their quality with silhouette numbers, for which we use the definition given in [15]. Silhouette numbers for individual simulations can range between $-1$ to 1, with positive values meaning that the cluster the simulation has been assigned to by the SOMs is the optimal one in the given classification and negative values meaning that the simulation would be better places in another cluster. Values near zero mean that the simulation is situated in space between two or more different clusters. The higher the silhouette numbers of all the simulations are overall, the better the classification found by the SOMs is. We evaluate the overall performance of the SOM classifications by average and median silhouette numbers, along with the proportion of negative silhouette numbers. Experimenting with the feature vectors defined above we found that the ones based on infection rates within districts and the district $\Lambda$ measures exhibited the highest average and median silhouette numbers at more than 0.8 and the lowest proportion of the negative silhouette numbers at less than 0.1, resulting in most easily recognizable clustering behaviours in the PCA visualisation. All the other classifications hardly reach average and median silhouette numbers of 0.7 at maximum, and barely recognizable clusters in the PCA visualisations.

In the following subsections we present the results of this procedure in the order given above. It should be noted that we are only interested in the large scale behaviour of the system, and so we use relatively simple methods as the PCA and SOMs to make our classifications, as opposed to more involved methods. Since the classification results are not interesting themselves, we assign them to an appendix, but in short, they can be characterised as follows: Infection rate and $\Lambda$ classifications form easily identifiable clusters, while the compliance classification appears to have two clusters touching each other, while the silhouette numbers suggest an optimum clustering with three SOM neurons. The economic and regulation classifications consist of bundles of filamentary structures that are not easily clustered. Next we take a closer look at the infection rate, regulation and $\Lambda$ classifications, while ignoring the economic and compliance classifications. As stated above, our goal is to find easily identifiable phases, not to shift through all the possible fine structures present in the data we generated as part of this parameter survey.

## Results

### The classification by infection rates and $\Lambda$ measures

**Fig. 1:**
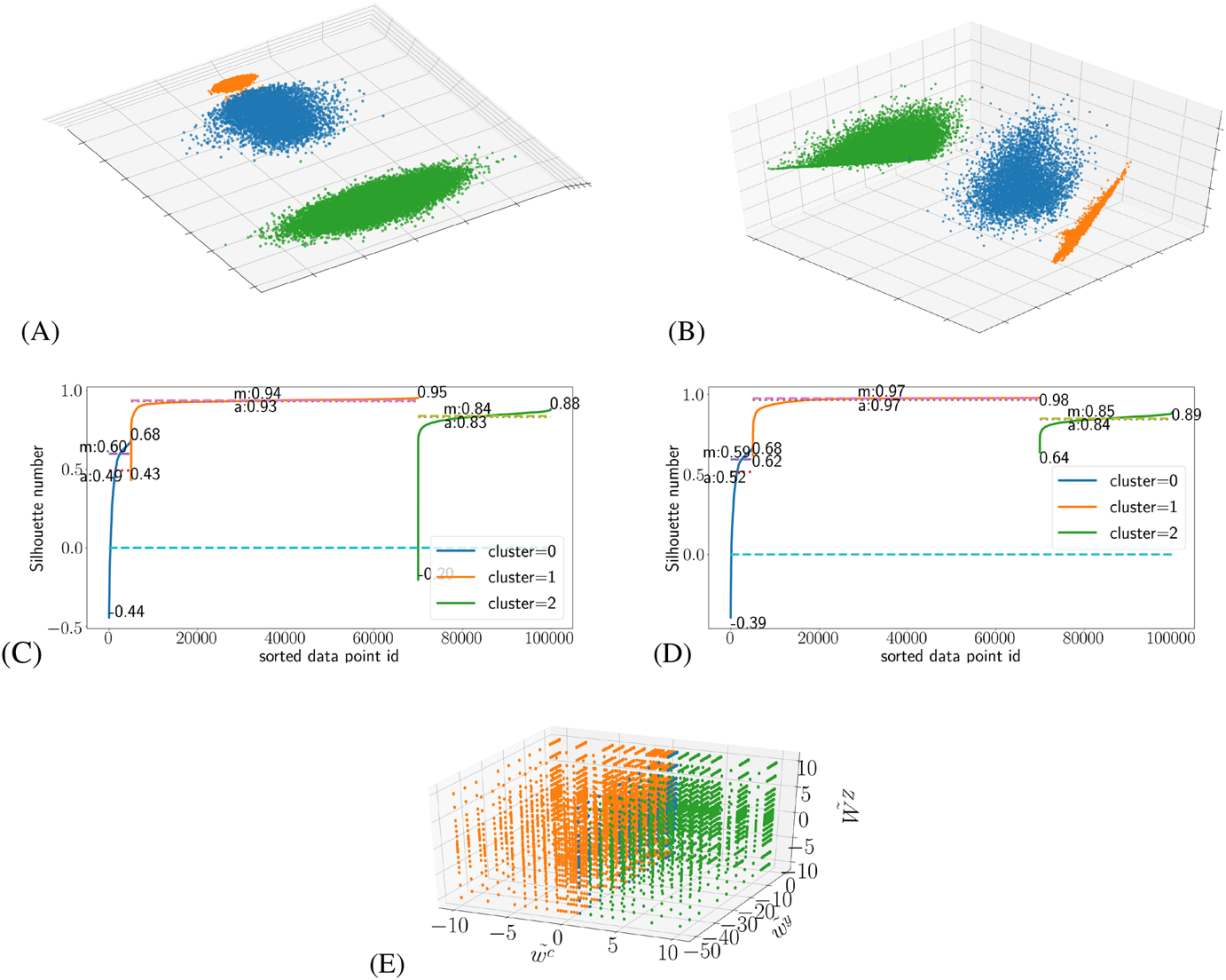
The SOM classifications based on average infection rates and $\Lambda$ measure with three SOM neurons: **A** Exhibits the distribution of the SOM clusters as arranged by PCA for the former and (**B**) for the latter. **C** and **D** show the corresponding silhouette profiles of the classifications., which are constructed by arranging the simulations in the SOM assigned clusters to ascending order according to their silhouette numbers, and then plotting their silhouette number by clusters. In the profiles we have marked the maximum, minimum, average (*a*) and median (*m*) silhouette numbers of all three clusters. **E** Shows a three dimensional distribution of the clusters in the value parameter space for the infection rate classification, when the fourth dimension $\tilde{W}^Y$ is collapsed into the others

As shown in the appendix, the infection rate and $\Lambda$ classifications using SOMs produce consistently the highest quality results as judged by the silhouette numbers, when compared with other classification schemes used. In this section we study why this is the case. The easiest way to see this is by taking a closer look to the classifications using three SOM neurons. The top panels of Fig. 1 illustrate this case in the PCA space, while the middle panels show the silhouette profiles.

The positioning of these clusters in the value parameter space is shown in panel (E) of Fig. 1, in which the four dimensional parameter space is projected into a three dimensional space by collapsing $\tilde{W}^Y$ to the other dimensions. Since the clusters are very similar in shape in both the infection rate and $\Lambda$ classifications, only the former is shown. Similarly, it makes little difference if $\tilde{w}^y$ were collapsed instead of $\tilde{W}^Y$.

Panel (A) of Fig. 1 shows that there are a total of three different clusters of varying densities that SOM has identified correctly. In the order of increasing density we have cluster 2 coloured green, cluster 0 coloured blue and cluster 1 coloured orange. Similarly, in the case of $\Lambda$ classification shown in panel (B) of Fig. 1 there are three clusters, two of which look like wings and a third of which is less well defined in shape. In the value parameter space, as shown in Fig. 1(E), we see that these clusters mostly occupy the spaces defined by their position on the $\tilde{w}^c$ axis: Cluster 1 consists of the simulations on the left side of the $\tilde{w}^c = 1$ plane, while cluster 2 takes most of the right hand side of the the $\tilde{w}^c = 1$ plane. Cluster 0 consists of points in the $\tilde{w}^c = 1$ plane.

The silhouette profile of the infection rate classification shows that judging by the mean and median silhouette numbers, cluster 1 is probably the best defined of these clusters, followed by cluster 2 and then cluster 0. The results for the $\Lambda$ measure classification are almost identical to the infection rate classification in the value parameter space (and so we only display the infection rate classification results in panel (E) of Fig. 1), while the silhouette numbers indicate that the all the clusters are generally much better defined in the $\Lambda$ classification in comparison to their equivalents in the infection rate classification, especially when the minimum silhouette numbers are considered.

Having established these clusters with the use of SOM, we have to now determine their physical meaning. To that end we calculated some statistics on the clusters, which we show in Table 2 for the infection rate classification and in Table 1 for the $\Lambda$ classification. We calculated the mean value parameters of all the agents and the mean infection rates of the geographic cells from each of the simulations, and displayed the minimum. average and maximum values of those in the Tables. Also included is the variance of the means in the clusters. The tables show the ranges of the value parameters and the quantities that the classification is based upon. The results of [1] identified two main behavioural types in the BTH-SEIRS model: the regular and fast spreading wave fronts with negative $\tilde{w}^c$ and slow and chaotic spreading patterns with positive $w^c$, which clearly correspond to clusters 1 and 2 of the infection rate and $\Lambda$ classifications, respectively. Cluster 0 is the transitional cluster between the other types, with a behavioural type that we called broken wave.

**Table 1:** Cluster statistics with $\Lambda$ classification

Cluster	Variable	Min	Mean	Max	Var
0	$\Lambda$	$4.03 \times 10^{-2}$	0.11	0.22	$1.10 \times 10^{-3}$
0	$\tilde{W}^Y$	− 50.10	− 10.65	− 0.15	225.48
0	$\tilde{W}^Z$	− 10.10	$2.04 \times 10^{-4}$	10.10	21.22
0	$\tilde{w}^y$	− 50.01	− 10.65	− 0.24	225.49
0	$\tilde{w}^c$	0.99	1.00	1.01	$1.16 \times 10^{-5}$
1	$\Lambda$	$1.20 \times 10^{-2}$	$1.46 \times 10^{-2}$	$3.35 \times 10^{-2}$	$3.14 \times 10^{-6}$
1	$\tilde{W}^Y$	− 50.10	− 10.65	− 0.15	225.50
1	$\tilde{W}^Z$	− 10.10	$1.89 \times 10^{-5}$	10.10	21.22
1	$\tilde{w}^y$	− 50.01	− 10.65	− 0.24	225.50
1	$\tilde{w}^c$	− 10.02	− 2.5	0.76	10.13
2	$\Lambda$	0.29	0.35	0.42	$2.42 \times 10^{-4}$
2	$\tilde{W}^Y$	− 50.10	− 10.65	− 0.15	225.50
2	$\tilde{W}^Z$	− 10.10	$7.92 \times 10^{-6}$	10.10	21.22
2	$\tilde{w}^y$	− 50.01	− 10.65	− 0.24	225.49
2	$\tilde{w}^c$	1.99	5.25	10.16	7.48

**Table 2:** Cluster statistics with infection rate classification

Cluster	Variable	Min	Mean	Max	Var
0	$y_i$	$3.75 \times 10^{-2}$	$5.97 \times 10^{-2}$	$7.41 \times 10^{-2}$	$4.48 \times 10^{-5}$
0	$\tilde{W}^Y$	− 50.10	− 10.69	− 0.15	226.03
0	$\tilde{W}^Z$	− 10.10	$-1.27 \times 10^{-2}$	10.10	21.24
0	$\tilde{w}^y$	− 50.01	− 10.68	− 0.24	225.94
0	$\tilde{w}^c$	0.99	1.00	1.01	$1.16 \times 10^{-5}$
1	Inf	$6.68 \times 10^{-2}$	$7.55 \times 10^{-2}$	$7.83 \times 10^{-2}$	$4.18 \times 10^{-7}$
1	$\tilde{W}^Y$	− 50.10	− 10.65	− 0.15	225.46
1	$\tilde{W}^Z$	− 10.10	$1.01 \times 10^{-3}$	10.10	21.22
1	$\tilde{w}^y$	− 50.01	− 10.65	− 0.24	225.46
1	$\tilde{w}^c$	− 10.02	− 2.50	1.01	10.14
2	Inf	$1.77 \times 10^{-3}$	$1.41 \times 10^{-2}$	$4.43 \times 10^{-2}$	$1.96 \times 10^{-5}$
2	$\tilde{W}^Y$	− 50.10	− 10.65	− 0.15	225.49
2	$\tilde{W}^Z$	− 10.10	$2.68 \times 10^{-6}$	10.10	21.22
2	$\tilde{w}^y$	− 50.01	− 10.65	− 0.24	225.50
2	$\tilde{w}^c$	1.00	5.25	10.16	7.48

Observing the results shown in Table 1 we can also see that Cluster 2 has the slowest spreading epidemic and cluster 1 the fastest. These result in correspondingly higher infection rates in the latter, as shown by Table 2. The value parameters are very similar in all the clusters with the exception of the compliance. Clusters 2 and 0 have always positive compliance, while cluster 1 has mostly negative compliance. All value parameters other than compliance range the whole spectrum of allowed values in all the clusters, which shows definitely that compliance determines whether the chaotic pattern manifests itself in a given simulation run. The results on the value parameters on Table 2 are numerically almost identical to those of 1, with the only substantial exception being the minimum compliance value, which is 1.99 in the $\Lambda$ classification and 1.00 in the infection rate classification. In any case, results this similar suggest that both classifications deal with the same underlying reality, when it comes to the evolution of the simulated epidemic.

Two things should be noted at this point about the effects of increasing or decreasing the number of SOM neurons used to make the infection rate classification, which also apply to the $\Lambda$ measure classification:

1. If only two neurons were used to make the classification, clusters 0 and 1 (and their equivalents in the $\Lambda$ classification) will merge into one, while cluster 2 would remain effectively unchanged, which is the expected result judging from Fig. 5.
2. If more than three neurons are used to make the classification, clusters 1 and 2 will remain unchanged for the most part, while cluster 0 will be divided into as many new clusters as the neuron count allows the SOM to find.

These facts indicate that the transitional state from the wave spreading pattern to the chaotic pattern represented by cluster 0 is very complex, with great deal of local variation according to the value parameters used in each simulation. It is noteworthy that the density of the three clusters seems to decrease in the PCA visualisation as their regularity increases, which is perhaps to be expected as PCA organizes the simulations according to their contribution to the variance between the simulations. At least intuitively one would expect more variability in the chaotic spreading pattern than the wave like pattern.

**Fig. 2:**
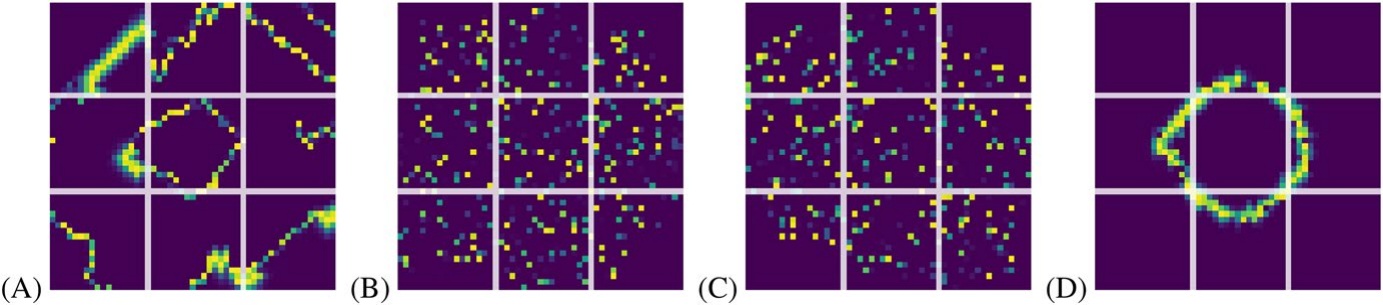
Representative visualisations of the simulations near the centroids of the clustersWith two examples for cluster 0 for purpose of demonstrating the effects of small differences at $\tilde{w}^c = 1$ point. . (A): cluster 0 when $\tilde{w}^c = 0.99$, $\tilde{w}^y = \tilde{W}^Y = -10$ and $\tilde{W}^Z = 0$, (B) The same as (A), except $\tilde{w}^c = 1.01$ (C): cluster 2 ($\tilde{w}^c = 5.2$, $\tilde{w}^y = \tilde{W}^Y = -10.7$ and $\tilde{W}^Z = 0$), (D): cluster 1 ($\tilde{w}^c = -2.5$, $\tilde{w}^y = \tilde{W}^Y = -10.7$ and $\tilde{W}^Z = 0$)

**Fig. 3:**
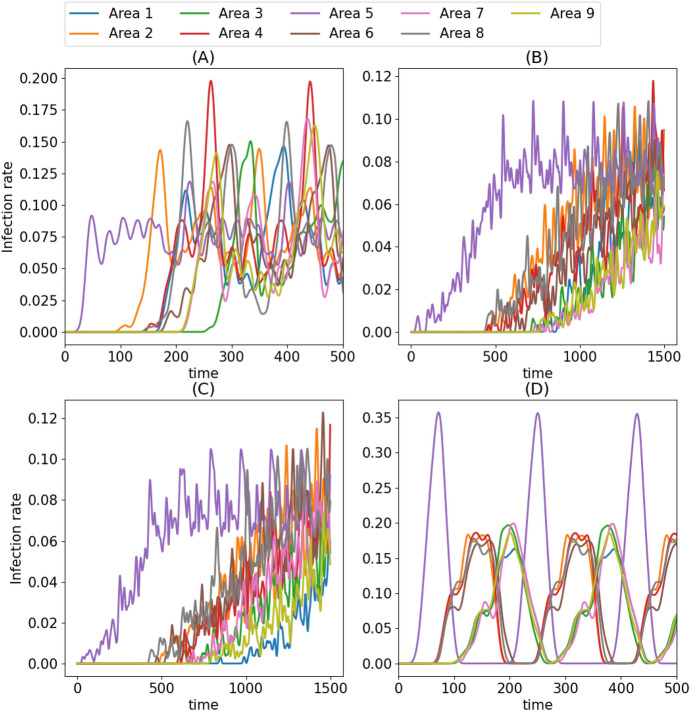
Infection rates of the simulations shown in in Fig. 2 , with matching panel labels

In order to demonstrate the behavioural types of the clusters found by SOM, we performed simulations in the vicinity of the centroids of the clusters in the value parameter space. Because $w^c_i$ must not have exactly the same numerical value as $w^x_i$, $\tilde{w}^c$ cannot have exact value of 1. That is why we chose to perform two simulations near this critical point for the cluster 0 and display both of them in order to demonstrate the effect of the compliance value parameter. Fig. 2 visualizes the results: Panels (A) and (B) represent cluster 0 at different sides of the $\tilde{w}^c = 1.0$, while panels (B) and (C) represent clusters 2 and 1, respectively. (A) shows a transitional state between chaotic and wave-like spreading types where the spreading pattern is still wavelike in form, but very irregular in comparison with the fully wave-like spreading pattern shown in Panel (C). Panel (B) in contrast shows spreading pattern where some regularity remains in the form of small pockets of very irregular wave-like fronts, but very similar to the fully chaotic spreading patterns shown in panel (D).

The time-evolution of the district infection rates of these simulations, averages of which the present classification is based on, are depicted in Fig. 3. The wave-like cluster 1 exhibits naturally very regular periodic behaviour, while the chaotic cluster 2 has not even fully settled into a regular periodic pattern. The behaviour of the transitional cluster 0 can be very similar to the chaotic or wavelike clusters depending on which side of the $\tilde{w}^c = 1$ plane they happen to be on. The infection rates found in chaotic side of cluster 0 are only slightly more elevated when compared with the fully chaotic cluster, with the exception of area 1, which has significantly lower infection rates after $t \approx 750$. Due to the chaotic nature of the spreading in chaotic cluster, however, this is a situation which may have arisen purely by chance. The wave-like side of cluster 0 is much more different from the fully wave-like cluster 1, with the infection rates being very regular in the latter, and irregular in the former.

The reason for the transition from wavelike spreading patterns to chaotic ones at $\tilde{w}^c = 1$ can be found in Eq. 7, where $(\tilde{w}^c - 1)^{-1}$ appears as a coefficient. In the simulations the value parameters are randomized for each population agent so that $0.9 \le \tilde{w}^c \le 1.1$ in the vicinity of the $\tilde{w}^c = 1$ point. this means that $(\tilde{w}^c - 1)^{-1}$ can have both positive and negative values, and that those values have very large absolute values. In fact,

13 $$\begin{aligned} |(\tilde{w}^c - 1)^{-1} |\ge 10 \end{aligned}$$

always. The end result is that in this limiting case all the population agents are either doing their utmost or very little, if anything at all, to mitigate the spread of the epidemic, and it is random which behavioural type any given agent adopts. In the simulations this manifests as the massive difference in behaviour shown in panels (A) and (B) of Fig. 2.

$\tilde{W}^Z > 0$ implies the hypothetical scenario in which governments would like to have their economy harmed by their own regulations, which is very unlikely. Therefore, in the appendix we show SOM classification using only simulations with $\tilde{W}^Z < 0$ as an additional experiment. The results for the infection rate and $\Lambda$ feature vectors are striking in that in these halved parameter spaces there are three clear clusters to be found that clearly correspond to the three main behavioural types of the model detailed above. In short in this limited space the complications brought by wavelike portion of cluster 0 disappear.

### Uncertainty test

In order to test our value parameter randomisation scheme and the stability of our model under multiple repetitions, we chose to perform 100 iterations of the model near three points representing the centroids of the clusters $0-2$ with the same randomisation scheme that we used to generate the data for the classification programs. The value parameters used are the same as used for clusters 1 and 2 in Figs. 2 and 3, but for cluster 0 we used only one point with $\tilde{w}^c = 1$, $\tilde{w}^y = \tilde{W}^Y = -10.6$ and $\tilde{W}^Z = 0$, since only one point in each was needed for the purposes of this uncertainty test. From this independent dataset we calculated statistics on the infection rates ($y_i$), the reduction of the economic activity ($x_i$) and the dynamic compliance of the population agents, the restrictions put in place by the authority agents ($X_i$) and the $\Lambda$ factor. The results of this test are given in Table 3. The randomisation scheme naturally causes some variations, but the variances are very small.

**Table 3:** Results of the uncertainty test

Cluster	Variable	Min	Mean	Max	Var
0	$y_i$	$4.9 \times 10^{-2}$	$5.92 \times 10^{-2}$	$6.59 \times 10^{-2}$	$7.28 \times 10^{-6}$
0	$x_i$	0.62	0.70	0.74	$4.41 \times 10^{-4}$
0	$c_i$	0.43	0.53	0.61	$1.7 \times 10^{-3}$
0	$X_i$	0.12	0.17	0.25	$7.34 \times 10^{-4}$
0	$\Lambda$	$9.72 \times 10^{-2}$	0.14	0.21	$3.05 \times 10^{-4}$
1	$y_i$	$7.72 \times 10^{-2}$	$7.78 \times 10^{-2}$	$7.84 \times 10^{-2}$	$9.15 \times 10^{-8}$
1	$x_i$	0.23	0.27	0.33	$4.31 \times 10^{-4}$
1	$c_i$	$-5.71 \times 10^{-2}$	$-3.95 \times 10^{-2}$	$-2.48 \times 10^{-2}$	$5.3 \times 10^{-5}$
1	$X_i$	0.25	0.31	0.39	$7.61 \times 10^{-4}$
1	$\Lambda$	$1.45 \times 10^{-2}$	$1.59 \times 10^{-2}$	$1.76 \times 10^{-2}$	$5.04 \times 10^{-7}$
2	$y_i$	$2.24 \times 10^{-2}$	$2.69 \times 10^{-2}$	$3.22 \times 10^{-2}$	$4.42 \times 10^{-6}$
2	$x_i$	0.51	0.57	0.63	$6.32 \times 10^{-4}$
2	$c_i$	0.35	0.40	0.47	$6.35 \times 10^{-4}$
2	$X_i$	0.15	0.17	0.18	$4.77 \times 10^{-5}$
2	$\Lambda$	0.31	0.35	0.37	$1.68 \times 10^{-4}$

### Classification by dynamic compliance $c_i$

**Fig. 4:**
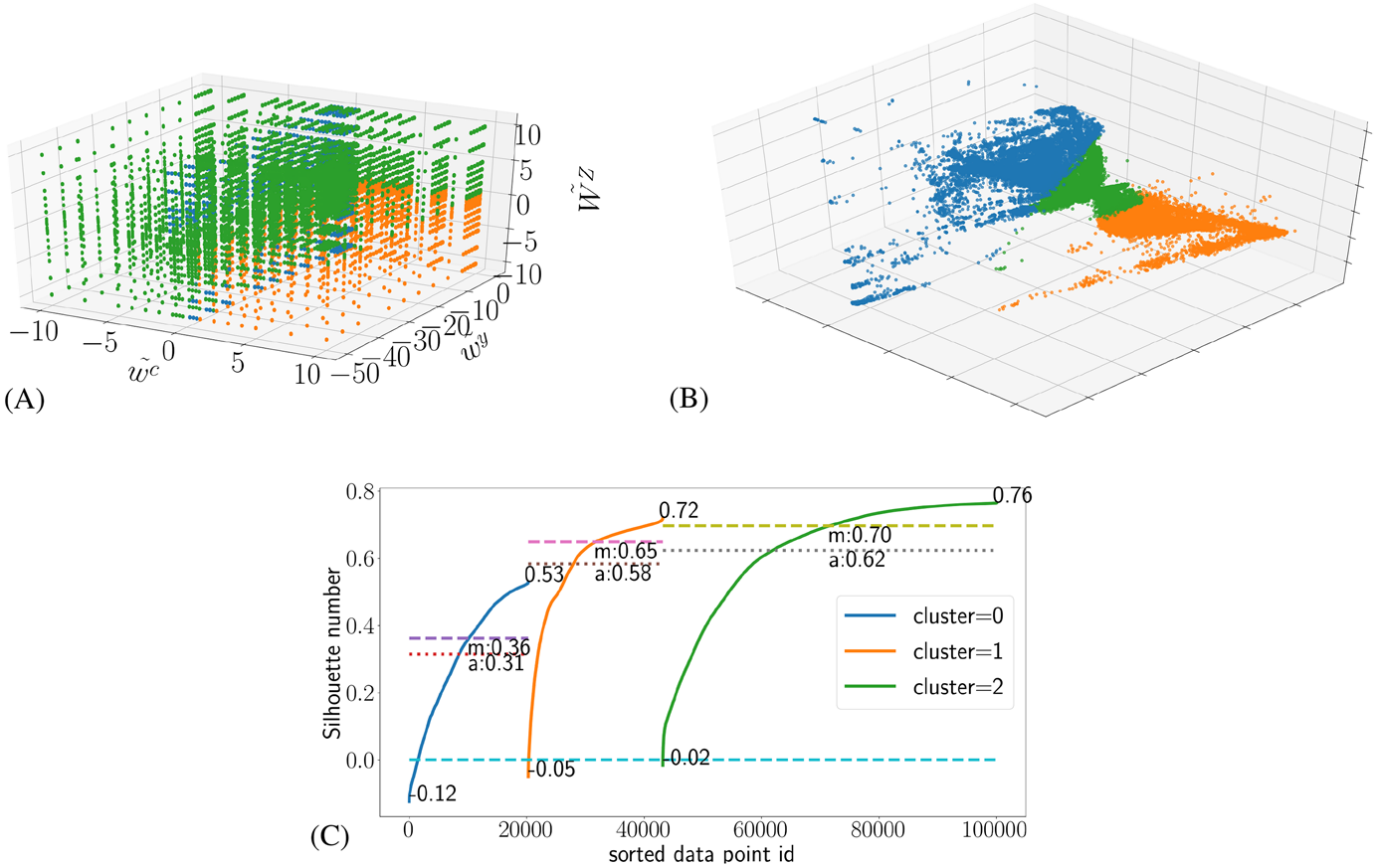
The classification using a three neuron SOM with the average dynamic compliance as the feature vector. **A** The classification in the value parameter space. **B** The same in PCA space. **C** The silhouette profile

**Fig. 5:**
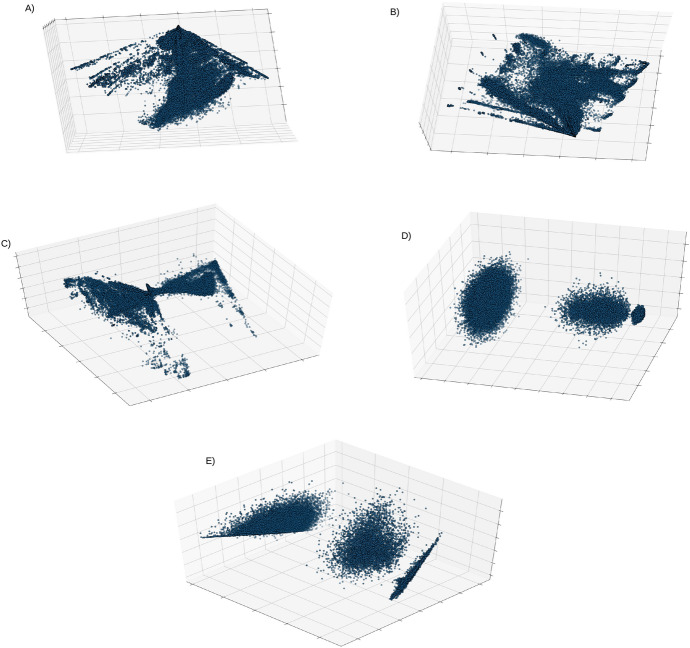
The PCA profiles of the feature vectors constructed form: **A** Average economic activity. **B** Government regulations. **C** Dynamic compliance. **D** Infection rates. **E** The $\Lambda$ measure

**Fig. 6:**
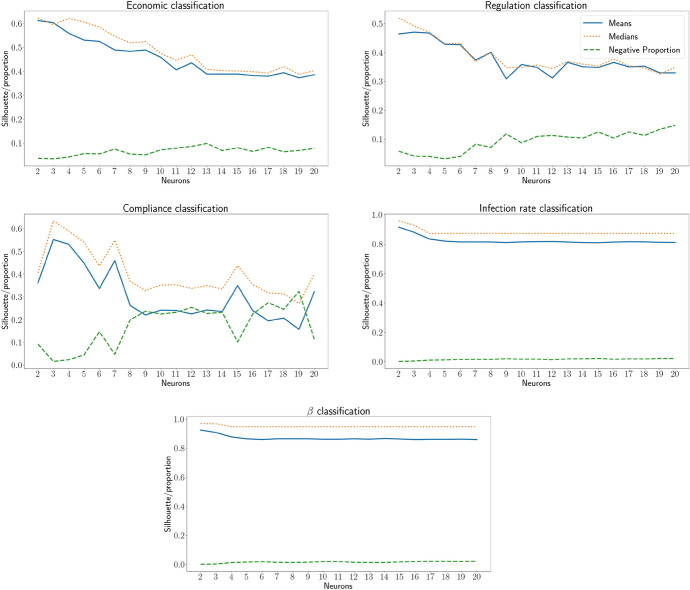
The mean and median silhouette numbers, along with the proportion of negative silhouette numbers when SOMs are used to classify the simulations according to the feature vectors visualised in Fig. 5

Judging from Figs. 5 and 6 in the appendix, the dynamic compliance classification seems most promising for closer analysis. While the shape of the PCA visualisation would suggest two clusters touching each other, the silhouette plot shows that three neuron SOM classification has the maximum average and median silhouette numbers, and the minimum proportion of negative silhouette numbers. The silhouette numbers are not as great as for the infection rate and $\Lambda$ classifications, but nonetheless we choose to show the results of this optimum classification in Fig. 4, which shows the identified clusters in the value parameter space (A) and the PCA visualisation (B), along with the silhouette profile (C).

The PCA visualisation shows two main clusters 0 and 1, with cluster 2 forming a bridge between them. In the value parameter space we can see that cluster 2 encompasses most of the $\tilde{w}^c < 0$ and $\tilde{W}^Z > 0$ sides of the value parameter space, leaving the $\tilde{w}^c > 0$ and $\tilde{W}^Z > 0$ quarter mostly for cluster 1. Cluster 0 seems to mostly consist of points near the $\tilde{w}^c = 0$ plane in Fig. 4. Cluster 2 seems best defined in terms of the silhouette clusters, followed by cluster 1 and then cluster 0. While the silhouette numbers are not even remotely as high as those found in the infection rate and $\Lambda$ classifications on any measure, they are at least mostly positive, in contrast to the transitional cluster in those classifications.

In order to make more sense of the SOM classification, we repeated the same statistical analysis we did for the infection rate and $\Lambda$ classifications and show the results in Table 4. In addition to the compliance $c_i$ we also display its components $X_i$ and $x_i$. It turns out that cluster 0 has the lowest compliance value on all measures (minimum, mean and maximum), while cluster 1 is revealed as the one with the highest compliance. Cluster 2 is then left with the average compliance values. With an average $\tilde{w}^c$ of $-0.16$ we can confirm the observation on the position of the cluster 0 we made visually from Fig. 4, although some points in cluster 0 can have as low values as $-5.01$ and as high as 1.01. All other value parameters of the points in cluster 0 run their whole allowed ranges, but their average values suggest that there is greater variability in the distributions of the clusters in the value parameter space in this classification than in the infection rate or $\Lambda$ classifications. For example, $\tilde{W}^Z$ has on average the value of 1.52, which is higher than the average of about 0 one would expect if the distribution of cluster 0 was symmetric with respect to $\tilde{W}^Z$. Similar asymmetries can naturally be seen in the statistics of cluster 2 as well: while all the value parameters span their whole allowed ranges, as one would expect from Fig. 4, there averages do not fall exactly near the values they would be if they were symmetric in terms of value parameters. This reflects the fact that clusters 0 and 2 intermingle in the same regions of the parameter space. Cluster 1 in turn has somewhat more clearly defined position, though it is surprising to see that $\tilde{W}^Z$ can have as high values as 5.09, when from Fig. 4 one would expect $\tilde{W}^Z < 0$ for all points in cluster 1.

**Table 4:** Cluster statistics with compliance classification

Cluster	Variable	Min	Mean	Max	Var
0	$c_i$	− 0.98	− 0.22	0.21	$2.42 \times 10^{-2}$
0	$X_i$	0.00	0.42	1.00	$6.58 \times 10^{-2}$
0	$x_i$	0.00	0.16	0.80	$2.74 \times 10^{-2}$
0	$\tilde{W}^Y$	− 50.10	− 17.91	− 0.15	295.01
0	$\tilde{W}^Z$	− 10.10	1.52	10.10	25.59
0	$\tilde{w}^y$	− 50.01	− 6.89	− 0.24	155.36
0	$\tilde{w}^c$	− 5.01	− 0.16	1.01	0.79
1	$c_i$	− 0.49	0.27	0.80	$2.22 \times 10^{-2}$
1	$X_i$	0.00	0.20	0.96	$3.33 \times 10^{-2}$
1	$x_i$	$8.19 \times 10^{-2}$	0.62	1.00	$2.44 \times 10^{-2}$
1	$\tilde{W}^Y$	− 50.10	− 11.65	− 0.15	243.34
1	$\tilde{W}^Z$	− 10.10	− 2.42	5.09	10.55
1	$\tilde{w}^y$	− 50.01	− 10.21	− 0.24	218.03
1	$\tilde{w}^c$	0.99	4.68	10.01	8.52
2	$c_i$	− 0.58	$3.19 \times 10^{-3}$	0.30	$5.09 \times 10^{-3}$
2	$X_i$	0.00	0.24	1.00	$7.19 \times 10^{-2}$
2	$x_i$	$2.11 \times 10^{-4}$	0.23	1.00	$6.19 \times 10^{-2}$
2	$\tilde{W}^Y$	− 50.10	− 7.65	− 0.15	165.25
2	$\tilde{W}^Z$	− 10.10	0.43	10.10	20.59
2	$\tilde{w}^y$	− 50.01	− 12.17	− 0.24	246.15
2	$\tilde{w}^c$	− 10.02	− 1.83	10.02	21.46

The results for the government regulations $X_i$ and the reduction of economic activity by the population agents $x_i$ shown in Table 4 reveal some interesting details about the compliance classification. For example, we can see that the average regulations in cluster 0 are about twice as tough as in the other two clusters, 0.42 as opposed to 0.23 in cluster 2 and 0.20 in cluster 1. Also, the population agents in cluster 0 reduce their economic activity on average less than the population agents in the other clusters, and even the maximum reduction they make is only 0.80 compared with 1.00 reduction in the other clusters (Notably, 1 is the maximum possible value for $x_i$). This may explain why the compliance values are lower in cluster 0 than the others. Another noteworthy observation is that the average reduction of economic activity is almost three times as high in cluster 1 than in the other clusters, 0.62 vs 0.16 and 0.23, while the regulations in the same cluster turn out to be the lowest at 0.20, although they are not much lower than the ones in cluster 2 with average regulations of 0.24. When it comes to minimum and maximum values of $X_i$ and $x_i$, all the clusters are actually very similar, with the exception of the maximum regulations of cluster 0, which are significantly lower than the respective maximum regulations in other clusters. Other than that, the maximum values are at or close to the maximum possible value of 1, and the minimum values are at or very close to the minimum possible value of 0.

Why does the SOM algorithm distinguish these three clusters in the case of the compliance feature vector, and what does that mean in relation to the infection rates? It is quite clear that cluster 1 is distinguished as a separate cluster due to it having relatively low regulations and very high popular commitment to epidemic mitigation, leading to very high rates of compliance in comparison with other clusters. In the case of cluster 2, on the other hand, the average regulations and popular mitigation measures are very similar in value, which results in near zero average compliance. It is likely that cluster 2 contains simulations that have both high regulations and high compliance (e.g. $\tilde{W}^Z > 0$ and $\tilde{w}^c > 0$) and simulations with low regulations and low compliance (e.g. $\tilde{W}^Z < 0$ and $\tilde{w}^c < 0$), which brings about this outcome. The remaining Cluster 0 is characterised by very high average regulations and very low popular mitigation efforts, which singles it out as the one with the lowest compliance. From Table 4 we can see that average $\tilde{W}^Y$ is substantially lower for this cluster than the others, which may contribute to the higher regulations, and that the average $\tilde{w}^y$ is higher, which in turn may contribute to the lower willingness of the population agents to take epidemic mitigation measures, along with mostly negative $\tilde{w}^c$. In summary, cluster 0 can be thought as the cluster in which authorities put up high regulations, but the populations do not follow them, cluster 1 can be thought of as a cluster where the authorities put up relatively low regulations, but populations go above and beyond in their epidemic mitigation measures, and cluster 2 is the cluster where populations on average match their mitigation measures with the regulations. Comparing these results with the infection rate and $\Lambda$ classifications we see that the $\tilde{w}^c > 1$ part of the value parameter space with generally lower infection rates is made up of cluster 1 in the $\tilde{W}^Z < 0$ half and the rest is taken up by a quarter of cluster 2. The $\tilde{w}^c < 1$ part is taken up by rest of the cluster 2 and cluster 0.

## Conclusions

In this study we have explored the parameter space of our hybrid socio-economic epidemic BTH-SEIRS model[1] to investigate different behavioural phases of the model and transitions between them. This was done by wrapping two of the value parameters, $W^X$ and $w^x$, into others and varying the resulting value parameters $\tilde{W^Z_i}$, $\tilde{w^c_i}$, $\tilde{W^Y_i}$ and $\tilde{w^y_i}$ for all the agents in the vicinity of 50, 000 points of the value parameter space. The mapping of this parameter space required in total of 100,000 simulations. The vast set of data was analysed with SOM to classify the data, PCA to help visualize the feature vectors used in the SOM classifications, and silhouette numbers to evaluate the quality of the SOM classifications. The feature vectors of our classifications were based on the averages of the four main quantities tracked by the model namely, the infection rates, economic activity, government regulations and the popular compliance, together with a special $\Lambda$ measure we introduced to measure the propagation speed of the epidemic. These data analysis techniques are powerful in revealing the most typical patterns in the data, which is in the focus of this study.

We find that the most significant value parameter in terms of the epidemic spreading type is the one representing the compliance of the general populations $\tilde{w^c_i}$. Depending on this parameter value two main behavioural types of the model emerge: the wavelike spreading patterns with $\tilde{w^c_i} < 1$ and the chaotic spreading patterns with $\tilde{w^c_i} > 1$, while $\tilde{w^c_i} = 1$ marks the transitional state between these two. SOM revealed that the transition from one pattern to another is very complex in the parameter space, as judged by the fact that increasing the number of neurons in the SOM results in the transitional cluster being subdivided into more sub-clusters. The classifications based on the $\Lambda$ factor turned out to agree with the infection rate classifications very closely.

Our analysis revealed significant patterns also with regard to compliance of the simulated populations. In this case silhouette numbers suggested three neurons to be the optimal for SOM classification, while shape of the feature vector in PCA visualisation seemed more like two clusters colliding with each other. A closer statistical look at the compliance, popular epidemic mitigation measures and epidemic regulations by the authorities in the clusters of the three neuron classification found by SOM showed that they represent recognizable joint behavioural patterns by the population and authority agents. In one cluster, mostly confined to the quarter of the parameter space with $\tilde{w^c_i} > 1$ and $\tilde{W^Z_i} < 0$, the authority agents have relatively lax regulations, while the population agents make significant epidemic mitigation efforts, resulting in a high dynamic compliance on average. In the second cluster, situated in the parameter space between $\tilde{w^c_i} \approx -5$ and $\tilde{w^c_i} \approx -1$, the agents have opposite behaviours when compared with their counterparts in the first cluster, meaning high regulations and low mitigation measures, resulting in low compliance. Third and final cluster takes up most of the space in the outside of the quarter dominated by the first cluster, and consists of simulations in which the mitigation measures by the population agents match, on average, the regulations made by the authority agents, causing the dynamic compliance to have near zero average value. There is a reason to believe that this cluster then contains both simulations with matching low or high regulations and mitigation measures, which makes it the most widely spread of all the clusters in the value parameter space.

In sum the full parameter scan of the model yielded new insights into the behavior of the model, and confirmed our preliminary results in [1]. The emphasis in the present study was to investigate measures describing the spread of the epidemic, namely the speed of its propagation and the dynamic infection rate. We found out that the compliance of the population is the major determining value parameter—in compliant populations the epidemic spread significantly slower than in non-compliant ones. Using dynamic compliance as the feature vector we were also able to identify three clusters that correspond to different joint behavioural types of the population and authority agents in our model. It turns out that in most areas of the value parameter space the mitigation measures by the population agents actually match the regulations given to them by the authority agents and, given how dynamic compliance is defined, high compliance can only really occur when regulations are relatively low. Similarly, low compliance is only possible when regulations are high. In this case the value parameter depicting how much authorities care about economic activity seems to be most important determinant of the stringency of the regulations they put in place, and together with the compliance of the population it determines how keenly the populations follow the regulations.

## Figures and Tables

**Fig. 7:**
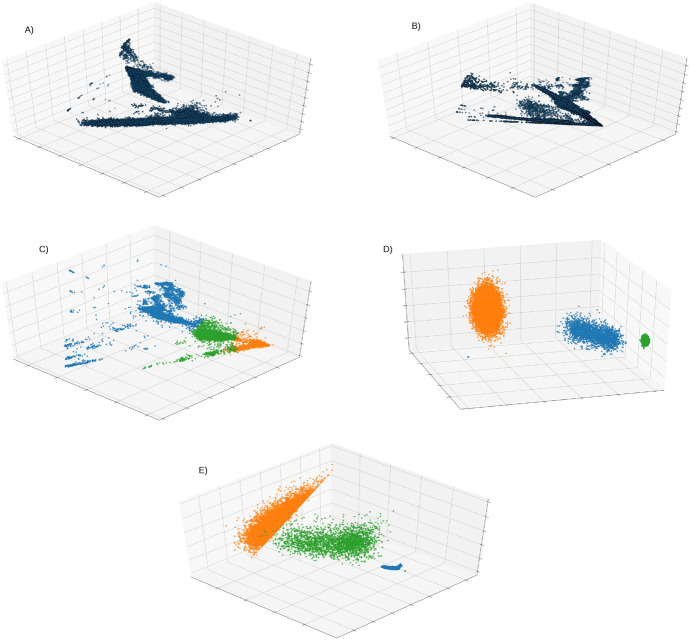
The PCA profiles of the feature vectors when applied only to the half of the simulations with $\tilde{W}^Z < 0$. The different feature vectors are presented in the same order as in Fig. 5. The $\Lambda$, infection rate and compliance classifications have been created using SOM with three neurons

## Data Availability

Data sets generated and the simulation codes utilized during the current study are available from the corresponding author on reasonable request.

## Appendix: PCA and silhuette results

Figure 5 shows the results of the PCA analysis of our data with these feature vectors. It turns out that the economic feature vector produces a shape in the PCA space that does not readily lend itself to classification, at least when judged with human eyes, as there is only one easily identifiable cluster with rather complicated structure and and with some filamentary features. This is also the case for the regulationfeature vector, although there are a lot more filamentary features. The feature vector made from the average dynamic compliance looks like two clusters of points touching each other, with interesting symmetrical filaments attached to each cluster. The feature vector constructed from the average infection rates seem the easiest to classify, as it takes the form of three easily distinguishable clustersof varying density. The $\Lambda$ measure feature vector, similarly, consist of three easily identifiable clusters, but the shapes of the clusters are very different from the infection rate feature vector: two of them are winglike, one has somewhat irregular shape.

It is clear from the PCA analysis that the feature vectors formed from the average governmental regulations, the infection rates and the $\Lambda$ measures are most amenable for computational classification. In this study we use SOM classification with 2–20 neurons, and evaluate the quality of these classifications with standard silhouette numbers [15]. Figure 6 shows the average and median silhouette numbers with different SOM neuron counts and feature vectors, along with the proportion of the simulations with negative silhouette numbers in each classification. As expected, the silhouette numbers of the classifications based on economic activity, dynamic compliance and government regulations are much lower than those based on infection rates and the $\Lambda$ measures, and generally higher proportions of the negative silhouette numbers.

Apart from the compliance classification, all the classifications show downward trends in median and average silhouette numbers as a function of the number of SOM neurons used, in addition to rising proportions of the negative silhouette numbers. However, with the the infection rate and the $\Lambda$ classifications the average and median silhouette numbers never dip below 0.8, while the economic and regulation classifications have maximum average and median silhouette numbers of about 0.6 and 0.5, respectively. While the proportions of the negative silhouette numbers stay just about below 0.1 for the economic classification, in the regulation classification they rise to numbers just above 0.1. The compliance classification also has the same general trends as the others, except that the average and median silhouette numbers rise significantly above their expected trends at neuron numbers 3, 7, 15 and 20. The proportions of the negative silhouette numbers also fall significantly at these neuron numbers. The silhouette numbers are the highest overall at neuron count 3, with median of about 0.63 and average of about 0.55. The proportion of the negative silhouette numbers also reach a minimum of approximately 0.2 at this point.

### Halving the parameter space: $\tilde{W}^Z < 0$

As noted in the main text, $\tilde{W}^Z < 0$ means that local authorities are happy to have their economies decline, which would be a very unusual attitude in the modern world. Because of this concern, we also applied our PCA and SOM procedures to the set of simulations with $\tilde{W}^Z < 0$ as an experiment. The results can be seen in Fig. 7, in which the feature vectors are represented in the same order as in Fig. 5, and with the infection rate, $\Lambda$ and compliance feature vectors classified by SOM into three clusters. The main effect of halving the value parameter space seems to be accentuating the differences between the different clusters in the infection rate and $\Lambda$ classifications (panels (D) and (E), respectively) and splintering in the other classifications. The splintering is most dramatically observed in the case of the economic feature vector (panel (A)), in which we see the emergence of up to three different clusters. Contrary to what one might expect, however, these clusters are not neatly recognized by the SOM method, and so we have chosen not to show any clustering for the halved economic activity classification. The the government regulation feature vector (panel (B)) on the other hand looks like multiple colliding rodlike structures in the halved parameter space, and we could not detect simple clusters in this case with SOMs either. The compliance feature vector (panel (C)) has a similar structure to the regulation feature vector (not surprising, given that dynamic compliance is defined in part by the regulations), except with only one rodlike structure that has suggestive clustering formations along its length. These SOM method can classify quite nicely with two or three neurons, of which we chose to visualise the three neuron classification, like in earlier. While not all the points may be classified intuitively, this visualisation makes it more plausible that three is indeed the optimal number of neurons to use for classifying the compliance feature vector.
